# NMR resonance assignment of a fibroblast growth factor 8 splicing isoform b

**DOI:** 10.1007/s12104-023-10132-8

**Published:** 2023-04-29

**Authors:** Bruno Hargittay, Konstantin S. Mineev, Christian Richter, Sridhar Sreeramulu, Hendrik R.A. Jonker, Krishna Saxena, Harald Schwalbe

**Affiliations:** 1 Institute for Organic Chemistry and Chemical Biology, Center for Biomolecular Magnetic Resonance (BMRZ), Johann Wolfgang Goethe University, Max-von-Laue-Str. 7, 60438 Frankfurt/Main, Germany; 2 Structural Genomics Consortium, Johann Wolfgang Goethe University, Max-von-Laue-Str. 15, 60438 Frankfurt/Main, Germany

**Keywords:** Fibroblast growth factor, FGF8b, FGF8, FGF, Structure, Dynamics, Motions, FGFR, Fibroblast growth factor receptor.

## Abstract

The splicing isoform b of human fibroblast growth factor 8 (FGF8b) is an important regulator of brain embryonic development. Here, we report the almost complete NMR chemical shift assignment of the backbone and aliphatic side chains of FGF8b. Obtained chemical shifts are in good agreement with the previously reported X-ray data, excluding the N-terminal gN helix, which apparently forms only in complex with the receptor. The reported data provide an NMR starting point for the investigation of FGF8b interaction with its receptors and with potential drugs or inhibitors.

**Supplementary Information:**

The online version contains supplementary material available at 10.1007/s12104-023-10132-8.

## Biological context

Fibroblast growth factors (FGFs) are hormones that bind to their corresponding receptors (FGFRs), which belong to the family of receptor tyrosine kinases. The formation of a tertiary complex of FGF, FGFR, and heparin activates various downstream cascades that regulate cell growth, cell differentiation, and proliferation (Ornitz and Itoh 2015, 2022). Impairment of these signaling pathways can lead to severe diseases, including cancer, which is why FGF-FGFR interactions have been subject to intense investigations in the past years.

The human FGF family encompasses 18 members, which are divided into subgroups based on the phylogeny. FGF8b belongs to the FGF8 subfamily, together with FGF17 and FGF18 (Itoh and Ornitz 2004), which all signal in a paracrine manner. The human FGF8 gene encodes four different splice isoforms, FGF8a, b, e, and f, which differ by the length of their N-terminal sequence that determines their receptor binding specificity (Crossley and Martin 1995; Gemel et al. 1996; Olsen et al. 2006). FGF8b is expressed in the epithelium and activates FGFR2c/FGFR3c splice isoforms and FGFR4 in the mesenchyme. It is known to play a major role in physiological processes, such as cellular proliferation and differentiation during embryonic development. Here, it is highly involved in mid-hindbrain development, but also limb-bud development and formation of the heart, ear, and eye (Crossley et al. 1996a, b; Meyers et al. 1998; Sun et al. 1999). FGF8b is also involved in different forms of cancer, e.g. prostate and breast cancer (Mattila and Härkönen 2007).

Several research groups have successfully carried out structural investigations in the field of FGFs. Most of these studies employ X-ray crystallography, revealing the structures of many FGF and FGFR members and shedding light on the binding specificity and complex assembly of FGF-FGFR complexes (Plotnikov et al. 2000; Schlessinger et al. 2000; Olsen et al. 2004; Herbert et al. 2013; Liu et al. 2017; Chen et al. 2018). The X-ray structure of FGF8b in complex with a two-domain construct of FGFR2c is also available (Olsen et al. 2006). Yet, substantial insight for FGF structural biology may be provided by NMR spectroscopy. In particular, the way in which FGFs and FGFRs recognize heparin molecules (Saxena et al. 2010) and the mode of action of low-molecular-weight medicines (Herbert et al. 2013) could be investigated. In our most recent work, we revealed how the D3 domain of FGFR3c interacts with SSR128129E (SSR), which is a small molecular compound that acts on FGFR as a pathway-specific allosteric inhibitor (Kappert et al. 2018). We found that the SSR binding site in the FGFR3c D3 domain overlaps with the binding site for FGF8b N-terminal groove-helix, specific for this particular growth factor. In this regard, it would be interesting to conduct NMR studies of FGF8b/FGFR interactions. Here, we present the almost complete NMR chemical shift assignment of FGF8b, which paves the way for future investigation.

## Methods and experiments

### Expression and purification of FGFR8b

The FGF8b construct used in this study includes amino acids Q23 to E186 of the full-length splice isoform of FGF8b (UniProt P55075), as used in previous studies (Olsen et al. 2006). The FGF8b gene was commercially synthesized and the sequence was inserted into a pKMProtG vector, containing an N-terminal His_6_-tag connected to a Protein G fusion protein and a tobacco etch virus (TEV) cleavage site. Due to the nature of the TEV cleavage site and the following NcoI-enzymatic cleavage site, the resulting FGF8b construct contained four additional amino acids at the N-terminus (^19^GAMG) that precede the native FGF8b sequence. The plasmid was used to transform the Shuffle® T7 competent *E.coli* cells (New England BioLabs). FGF8b was uniformly ^15^N- and ^13^C,^15^N-labeled by expressing the protein in M9 minimal medium containing 1 g/L ^15^NH_4_Cl, and 4 g/L ^13^C-D-glucose for double labeling and 100 µg/L ampicillin. Cultures were inoculated to an OD_600_ of ~ 0.1 and grown at 30 °C. Once cell cultures reached an OD_600_ ~ 0.6, they were put on ice for 5 min, prior to induction with 0.5 mM isopropyl-β-D-thiogalactoside (IPTG). Thereafter, expression of FGF8b was performed at 20 °C, 120 rpm for 18 h. Cells were harvested at 8,000 × g for 20 min using a Beckmann centrifuge with a JLA 8.1000 rotor. The pellet was suspended in 250 mL of buffer A (25 mM Tris pH 8, 500 mM NaCl, 2% glycerol, 1 mM β-mercaptoethanol), containing two protease-inhibitor tablets (cOmplete™, Roche, Germany). Cells were lysed in three cycles using a French press Microfluidics M-100P at a pressure of 15,000 PSI (pounds per square inch) under continuous cooling to 0 °C. The lysate was centrifuged at 4 °C and 35,000 × g for 45 min using a Beckmann centrifuge with a JA 20 rotor. Further purification steps of FGF8b included ion exchange chromatography (IEC), immobilized metal ion chromatography (IMAC), and size exclusion chromatography (SEC). First, the supernatant was passed through a syringe filter (pore size 0.22 μm, VWR) and then applied to a 5 mL HiTrap® Heparin High-Performance column (Cytiva, USA). FGF8b-ProteinG was eluted with buffer B (25 mM Tris pH 8, 2 M NaCl, 2% glycerol, 1 mM β-mercaptoethanol) using a step gradient (30% buffer B in 4 column volumes (CV), 32% buffer B until the baseline was reached, 100% B in 8 CV). Fractions containing the target protein were incubated with TEV-protease overnight at 4 °C. The cleaved fusion partner was removed using 3 × 5 mL HisTrap HP columns (Cytiva, USA). FGF8b was eluted with buffer C (25 mM Tris pH 8, 500 mM NaCl, 500 mM imidazole, 2% glycerol, 1 mM β-mercaptoethanol) using a linear gradient (100% buffer C in 10 CV), followed by a final buffer exchange via size exclusion chromatography (SEC) to buffer D (50 mM NaPi pH 6.5, 300 mM NaCl). Fractions containing the 19.4 kDa protein were concentrated using VivaSpin20 centrifugal concentrator devices with a 10 kDa MWCO (Sartorius, Germany). Purity was assessed by SDS-PAGE analysis after each purification step. Purity of FGF8b was further confirmed using mass spectrometry (MALDI-MS).

### NMR experiments

FGF8b samples were prepared in buffer (50 mM NaPi pH 6.5, 300 mM NaCl, 2% d_8_-glycerol, 5% D_2_O) with the addition of 0.5 mM TSP as an internal reference standard. The NMR samples (350 µL) were measured at 298 K on Bruker spectrometers (ranging from 600 to 950 MHz, equipped with the cryoprobes) in shaped tubes. Triple resonance spectra were acquired using the BEST-TROSY pulse sequences (Favier and Brutscher 2011; Solyom et al. 2013), and non-uniform sampling (NUS) of the indirect dimensions was applied, if required. The backbone and side chain chemical shift assignments of FGF8b were conducted using the standard triple resonance NMR experiments on uniformly ^13^C/^15^N-labeled samples at 298 K, with the assistance from 3D HCC(CO)NH and 3D NOESY-HSQC spectra. The list of recorded NMR spectra is provided in Table 1. Data were processed using TopSpin 4.1.1 software (Bruker BioSpin) and the manual assignment was conducted using Sparky (T. D. Goddard and D. G. Kneller, Sparky 3, University of California, San Francisco) and POKY software (Lee et al. 2021).

**Table 1:** List of the NMR experiments with experimental conditions

Experiments	Labeling	Time domain data size [points]			Spectral width [ppm]			ns	Delay time [s]	Spectrometer [MHz]	NUS %	Sample Conc [µM]
		T1	T2	T3	F1	F2	F3					
BEST-TROSY	^13^C/^15^N	256	1666		32.0 (^15^N)	13.2 (^1^H)		8	0.3	900	-	500
^1^H-^13^C-HSQC		256	1024		68.0 (^13^C)	16.3 (^1^H)		4	1.0	900	-	500
HNCACB		200	180	1666	64.0 (^13^C)	32.0 (^15^N)	13.2 (^1^H)	128	0.31	900	25	285
HN(CO)CACB		144	180	1666	64.0 (^13^C)	32.0 (^15^N)	13.2 (^1^H)	80	0.29	900	23	285
HNCO		128	192	1754	10.0 (^13^C)	32.1 (^15^N)	13.2 (^1^H)	24	0.3	950	25	285
HN(CA)CO		144	192	1754	10.0 (^13^C)	32.1 (^15^N)	13.2 (^1^H)	72	0.3	950	25	285
HNCA		160	160	1024	30.0 (^13^C)	32.0 (^15^N)	11.9 (^1^H)	24	0.3	600	25	500
H(CC)(CO)NH		160	160	1092	6.95 (^1^H)	32.0 (^15^N)	13.0 (^1^H)	32	1.0	600	25	500
(H)CC(CO)NH		160	160	1092	63.1 (^13^C)	32.0 (^15^N)	13.0 (^1^H)	32	1.0	600	11.4	500
NOESY-^15^N-HSQC		156	144	1370	11.4 (^1^H)	35.0 (^15^N)	14.0 (^1^H)	32	1.0	700	25	600
NOESY-^13^C-HSQC		232	180	1520	12.7 (^1^H)	36.8 (^13^C)	12.1(^1^H)	16	1.0	900	50	500

To obtain the ^15^N relaxation data we recorded the HSQC-based pseudo 3D experiments (Farrow et al. 1994). All data were measured at 600 MHz in an interleaved manner to exclude the influence of time-dependent processes. The following time points were used: 20, 40, 60, 100, 200, 400, 700, 1000, 1500 ms for T_1_ and 17, 34, 68, 136, 170, 204, 238 and 306 ms for T_2_. Heteronuclear {^1^H}-^15^N steady-state NOE was measured based on the two experiments recorded with and without the proton presaturation during the relaxation delay equal to 2 s. The obtained parameters were analyzed using the model-free approach and TENSOR2 software (Dosset et al. 2000).

## Extent of assignment and data deposition

### Chemical shift assignment

**Fig. 1:**
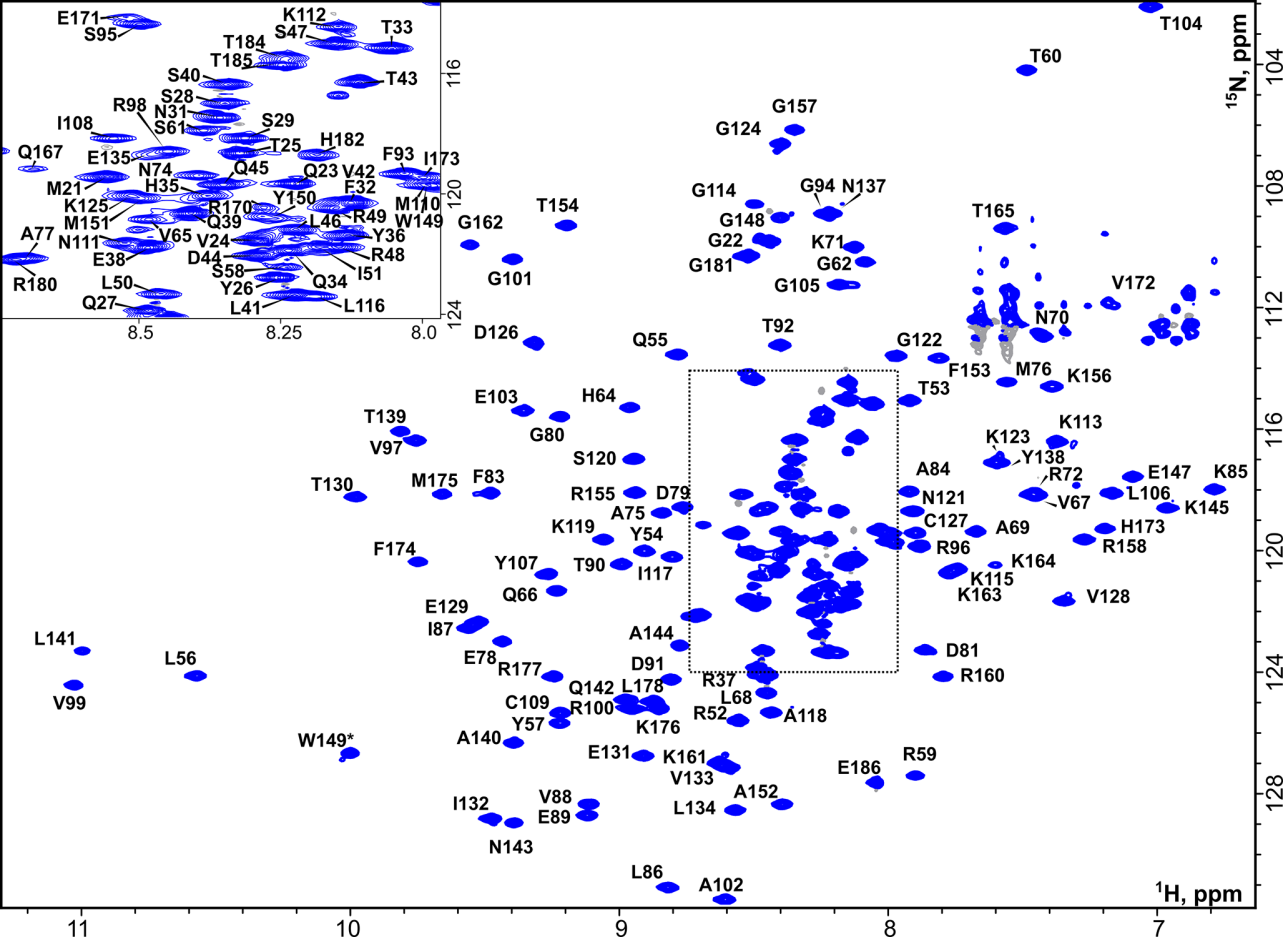
**NMR assignment of FGF8b.**^1^H,^15^N-BEST-TROSY spectrum of ^13^C/^15^N-labeled FGF8b, recorded at 298 K in 50 mM NaPi pH 6.5, 300 mM NaCl, 2% d_8_-glycerol, 5% D_2_O. Spectrometer working frequency was 900 MHz. Assignments of the backbone amide resonances are shown for each residue, an asterisk (*) denotes the signal of the protein side chain. Distorted signals in the top right corner correspond to the incompletely suppressed resonances of Asn/Gln NH_2_ groups

The FGF8b construct under investigation contained 168 residues including four prolines, suggesting that 163 cross-peaks are expected to be observed in ^1^H,^15^N-HSQC. We managed to assign 155 amide cross peaks, which is 95.6% of the possible amide signals (Fig. 1). Overall, we assigned 96.2% of the protein backbone (N, H, C, CA, HA) and 92.5% of the aliphatic side chain atoms. Signals of the aromatic side chains were not assigned. In addition, we identified the ^1^H and ^15^N resonances of eleven asparagine and glutamine sidechains, and ^1^Hε_1_ cross-peaks of tryptophan 149, based on the NOESY-HSQC connectivities. Amide signals for the first two amino acids of our construct and amino acids N136, S163, H168, Q169, and H183 were not found, most likely due to exchange-induced line broadening. However, for the residues G19, A20, N136, S163, Q169, and H183 we identified the chemical shifts of respective side chains. Thus, the only residue with no assignment is H168. The obtained chemical shifts were deposited to the Biological Magnetic Resonance Bank (BMRB; access number 51832).

### Analysis of chemical shifts

**Fig. 2:**
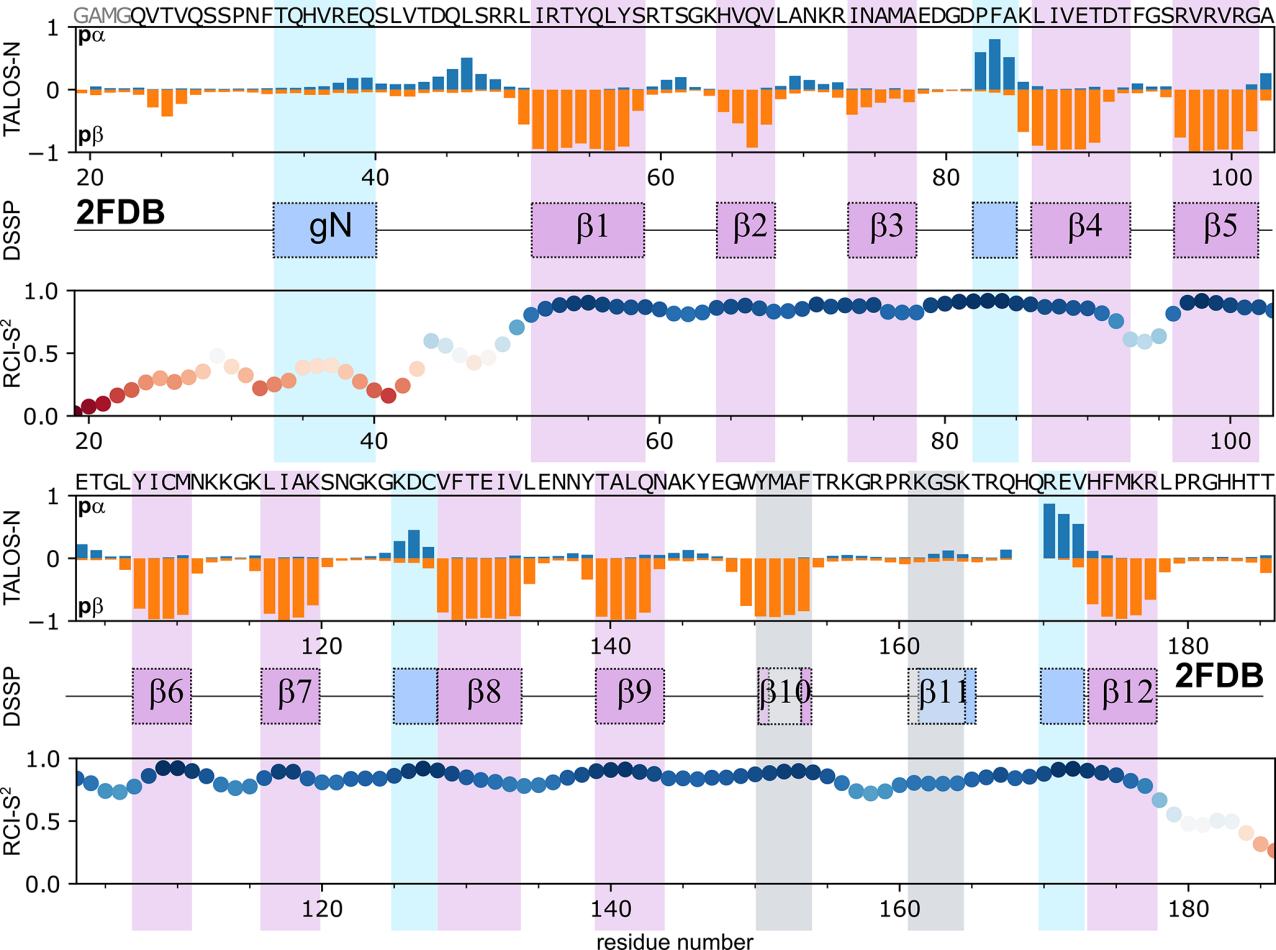
**Results of the FGF8b NMR chemical shift analysis**. The chemical shift-derived propensity of the secondary structure (**TALOS-N**, is orange and negative for the β-structure and blue or positive for the ɑ-helix); the secondary structure of FGF8b, according to the PDB ID 2FDB is shown for reference (**DSSP**, blue rectangles correspond to the helical structure and purple - to the extended conformation, grey rectangles for the strands β10 and β11 denote that these two elements are reported in the manuscript describing the X-ray structure (Olsen et al. 2006) but cannot be identified by the software); and the order parameters of backbone amide groups, calculated based on the random coil chemical shift index (**RCI-S**^**2**^), are shown as a function of residue number. Points are colored with respect to the RCI-S^2^ magnitude. The secondary structure shown is the consensus result of the DSSP (Kabsch and Sander 1983) and STRIDE (Heinig and Frishman 2004) packages. First four residues in the sequence of FGF8b are shown in gray, because they are the remnants of the protein expression tag and are absent in the native FGF8b and in the crystallization construct

**Fig. 3:**
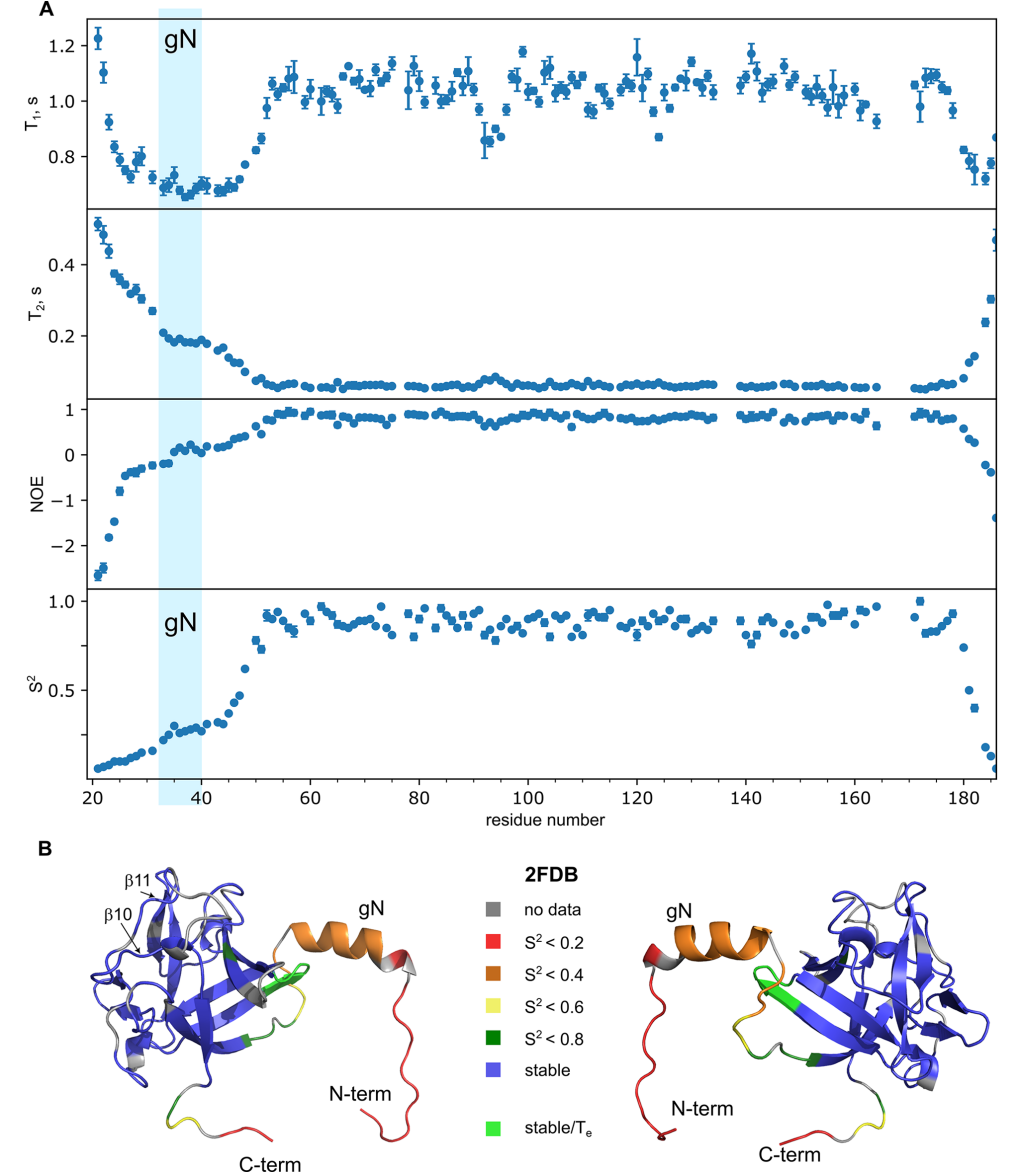
**Dynamics of FGF8b. A –** NMR relaxation parameters of ^15^N nuclei (T1, T2 and NOE) as well as the order parameters of amide groups, obtained through the model-free analysis of the relaxation data, are shown as a function of residue number in FGF8b. Blue area represents the position of a gN helix in PDB ID 2FDB. **B –** Spatial structure of FGF8b (PDB ID 2FDB, 180^o^ stereo view) is painted with respect to the backbone mobility (for the full set of raw data, see supplementary file). Color coding is provided in the legend. The first 13 N-terminal residues were not present in the PDB 2FDB and were added by computer modeling. Residues 91–95 are colored light-green due to the reliably detected presence of low-amplitude nanosecond motions (T_e_) that do not reduce substantially the corresponding order parameters

To check the consistency of NMR assignment and available 3D structure of FGF8b, we analyzed chemical shifts with TALOS-N software (Shen and Bax 2015) (Fig. 2). According to the literature, FGF8b placed in the complex with FGFR2c adopts a β-trefoil fold, consisting of 12 antiparallel β-strands (β1-β12) (Olsen et al. 2006). However, two of these strands, β10, and β11 are not identified in the PDB file by the conventional secondary structure analysis software, such as STRIDE (Heinig and Frishman 2004) or DSSP (Kabsch and Sander 1983), probably, due to the low resolution (Fig. 3B). In addition, the N-terminus of the protein forms a short α-helix, which was called groove helix or gN-helix, because its hydrophobic face is packed in the hydrophobic groove on the FGFR2c D3 domain (Fig. 2). In agreement with the X-ray data, NMR chemical shifts reveal the presence of eleven β-strands, however, the strand β3 is not very pronounced. Strand β11 cannot be identified in the NMR data, but, according to the analysis of PDB ID 2FDB, a short 3/10 helix is formed in the specified region, therefore the X-ray and NMR data are in agreement. Apart from the β-strands, our data reveal the presence of two pronounced short helical regions (83–85 and 171–173). Some helical propensity is also observed at the N-terminus (residues 35–50), however, it is clear that the gN helix (residues 33–40) is not formed. Analysis of this region with SSP software (Marsh et al. 2006) reveals the slight (up to 0.1) probability of helical structure. According to the random coil chemical shift index (Berjanskii and Wishart 2005), the N-terminus is disordered up to residue 50 and the beginning of strand β1. It is most likely that this part of the protein gets structured only upon the interaction with the FGFR2 D3 domain.

### Internal dynamics of FGF8b

We analyzed the NMR relaxation of ^15^N nuclei and processed the data using the model-free approach to detect local conformational dynamics (Supplementary file 1). The results are provided in Fig. 3. As one can see, overall the protein is dynamically stable in the region 50–178. At the N-terminus, the protein is highly flexible and experiences picosecond motions. However, one could identify two regions of distinct mobility. While the order parameters begin to decrease immediately prior to the first β-strand, there is a plateau on the S^2^ graph, observed for the residues 33–45, the region that includes the gN helix in the X-ray structure. Thus, while the helix gN is not formed, the corresponding region reveals a partially restricted mobility and can reside in some stabilized conformation for a certain share of the time. Interestingly, apart from the terminal regions, the nanosecond mobility is also observed in the D91-S95 loop (decreased T1 and the presence of nanosecond motions (T_e_) in the results of model-free analysis), which is adjacent to the helix gN in the X-ray structure. Apparently, the corresponding part of the molecule may interact with the loop, which could cause the observed stabilization.

In conclusion, here we report an NMR chemical shift assignment of human growth factor FGF8b. The assignment is almost complete and is in good agreement with the spatial structure of FGF8b reported previously. The only substantial difference is the presence of a gN helix, which is likely to be formed only upon the complex formation between the growth factor and its receptor. The quality of the data and completeness of the assignment allow exploiting our data in future studies of the receptor-ligand interaction mechanisms.

## Electronic supplementary material

Below is the link to the electronic supplementary material.

Supplementary Material 1

## Data Availability

NMR resonance assignments have been deposited to the BMRB under the accession code: 51832. NMR relaxation parameters and results of their analysis in TENSOR2 software are provided in supplementary file.
